# Masked Generative Light Field Prompting for Pixel-Level Structure Segmentations

**DOI:** 10.34133/research.0328

**Published:** 2024-03-26

**Authors:** Mianzhao Wang, Fan Shi, Xu Cheng, Shengyong Chen

**Affiliations:** ^1^ The Engineering Research Center of Learning-Based Intelligent System (Ministry of Education), Tianjin University of Technology, Tianjin 300384, China.; ^2^ Key Laboratory of Computer Vision and System (Ministry of Education), Tianjin University of Technology, Tianjin 300384, China.; ^3^ School of Computer Science and Engineering, Tianjin University of Technology, Tianjin 300384, China.

## Abstract

Pixel-level structure segmentations have attracted considerable attention, playing a crucial role in autonomous driving within the metaverse and enhancing comprehension in light field-based machine vision. However, current light field modeling methods fail to integrate appearance and geometric structural information into a coherent semantic space, thereby limiting the capability of light field transmission for visual knowledge. In this paper, we propose a general light field modeling method for pixel-level structure segmentation, comprising a generative light field prompting encoder (LF-GPE) and a prompt-based masked light field pretraining (LF-PMP) network. Our LF-GPE, serving as a light field backbone, can extract both appearance and geometric structural cues simultaneously. It aligns these features into a unified visual space, facilitating semantic interaction. Meanwhile, our LF-PMP, during the pretraining phase, integrates a mixed light field and a multi-view light field reconstruction. It prioritizes considering the geometric structural properties of the light field, enabling the light field backbone to accumulate a wealth of prior knowledge. We evaluate our pretrained LF-GPE on two downstream tasks: light field salient object detection and semantic segmentation. Experimental results demonstrate that LF-GPE can effectively learn high-quality light field features and achieve highly competitive performance in pixel-level segmentation tasks.

## Introduction

In recent years, the emergence of the metaverse has unveiled the rapid advancements in autonomous driving systems [1,2], as it possesses the capability to simulate diverse driving environments and sensor conditions. Pixel-level structure segmentation, a key computer vision task within autonomous driving systems, has attracted considerable attention, encompassing tasks such as salient object detection and semantic segmentation [3–6]. Such works can provide pixel-level predictions, facilitating autonomous driving systems in discerning object categories, shapes, and positions, effectively mirroring human visual perception. With the continuous improvement of deep learning technology [7–9], pixel-level structure segmentations are progressing toward intelligence and automation, placing a growing demand on advanced feature modeling methods [10–12]. Nevertheless, current feature modeling methods are markedly constrained by the inherent planar information in traditional two-dimensional (2D) imaging. In the real autonomous driving environment, the appearance information projected onto a single 2D plane by traditional imaging struggles to convey the 4D geometric cues necessary for describing the semantic relationships within a 3D scene and constructing a comprehensive feature space. This deficiency may result in erroneous segmentation outcomes, especially in situations with similar foreground and background appearances or when confronted with cluttered background information. Hence, acquiring comprehensive 4D geometric information and developing a robust feature modeling method for pixel-level structure segmentation tasks can significantly enhance the perception capabilities of autonomous driving while also reducing safety threats in complex environments.

Current research in acquiring rich 4D geometric information for pixel-level structure segmentations is mainly implemented using light field imaging technology [3,4,13,14]. Compared to other complex imaging technologies, light field imaging can simultaneously capture the distribution of light rays at every point along every direction in a single snapshot, creating a comprehensive record of how light behaves within a physical volume [5,13,15]. Utilizing the two-plane model [16], the light field can be parameterized as multiple views, denoted as *L* (*u*, *v*, *x*, *y*), observed from viewpoints arranged in a regular grid on a 2D plane. As a result, light field images not only encompass appearance information but also capture the geometric structure of the scene, incorporating both the 4D spatial and angular information of the light rays. In general, existing light field-based feature mining primarily focuses on developing complex modeling methods. As depicted in [6,14], the multi-stream modeling methods involve extracting appearance-related features from various views within the light field using pretrained models, such as ResNet [10] and ViT [11]. Subsequently, these features are fused to analyze the distinctive attributes of the light field. In contrast to the multi-stream approach, two-stream modeling methods [17,18] consist of a 2D RGB stream responsible for extracting appearance-related features from a single view or an all-focus image, in conjunction with a specifically designed task-oriented light field stream aimed at capturing the spatial-angular cues intrinsic to the light field. Ultimately, these two sets of features are fused to facilitate downstream tasks. Therefore, the wealth of 4D information contained within the light field can construct visual features significantly, contributing to the robustness of pixel-level structure segmentations.

However, current light field modeling methods fail to fully capitalize on the advantages of the 4D light field in pixel-level structure segmentation. Multi-stream modeling methods offer the distinct advantage of retaining the prior knowledge acquired during the pretraining phase, leading to improved accuracy and efficiency. However, an excessive emphasis on the RGB backbone may cause the model to prioritize appearance features over the inherent geometric structural information within the light field. While two-stream modeling methods can effectively extract geometric structural information from the light field, there exists a feature misalignment problem between the geometric structure and appearance, meaning they do not share the same feature space. This constraint limits the capacity for transferring visual knowledge through the light field. Consequently, the motivation of this paper is to develop a universal light field modeling method capable of extracting geometric structural information from the light field and aligning the multiple modal features into a unified feature space.

Recently, generative artificial intelligence (GAI) and masked image modeling (MIM) have achieved remarkable success in computer vision [19]. The vector-quantized generative adversarial network (VQGAN), as a generative model within GAI, possesses the capability to transform inputs into discrete feature representations [20,21]. These discrete feature representations, situated within a latent space, can generate realistic images, thereby equipping it with the ability to capture general visual cues. Meanwhile, MIM, serving as a pretraining paradigm in self-supervised learning [22,23], has the capability to learn data-related features from large-scale unlabeled datasets. Therefore, we can leverage these two technologies to develop a universal light field modeling framework. This framework can utilize MIM for self-supervised learning, allowing the neural network to extract rich geometric structural guidance. Additionally, it can employ GAI to align multiple modal features within a unified visual space. However, achieving this goal presents challenges. First, existing backbones for light field feature modeling often struggle to establish a connection between the geometric structure and the appearance within the light field [17,24,25]. This limitation makes it difficult to leverage VQGAN [21] for aligning these features, especially within a multi-scale feature space. Additionally, the full-training light field network increases the computational burden, further complicating the modeling of light field features. Consequently, designing a light field backbone to align multiple modal features and fuse them remains a challenge. Second, current MIM methods [22,23] require deployment on large-scale unlabeled data. However, the availability of datasets for light fields is limited. Furthermore, the reconstruction of the complete topological structure of the light field, rather than merely pixel reconstruction from a single view, remains largely unexplored at present. Designing a MIM framework based on light fields presents yet another significant challenge.

In this paper, we propose a universal light field feature modeling framework for pixel-level segmentations. This framework can simultaneously extract both appearance and geometric structural cues from light fields, aligning these features into a unified feature space. Specifically, the proposed method contains an overall light field backbone, termed generative light field prompting encoder (LF-GPE), and a generalized light field pretraining network, namely, prompt-based masked light field pretraining (LF-PMP). For LF-GPE, we initially freeze the patch embeddings from the central view of the light field, enabling them to extract multi-scale appearance features from 2D images without the need for parameter training. Subsequently, we employ a VQGAN to convert the epipolar plane views of the light field into semantic tokens, aligning them within a unified visual semantic space. Following this step, we utilize this aligned light field information as a generative prompting for semantic interaction. Ultimately, we merge the interacted light field features with appearance features in a multi-scale space. For LF-PMP, we begin by generating a mixed light field during the encoding phase, using two sets of randomly trained light fields. This mixed light field compels LF-GPE to extract a wealth of geometric structure information. Following the same setup as in LF-GPE, we maintain the frozen state of patch embeddings from the central view while fine-tuning the generative light field prompts, in addition to the semantic interaction and modulation modules. Finally, we perform multi-view light field reconstruction to recreate the original light field structures from the mixed input. In experiments, we perform pretraining on a collected mixed light field dataset and evaluate our approach on two downstream tasks: light field saliency object detection and semantic segmentation. Our simple and unified light field modeling approach achieves highly competitive performance in pixel-level segmentation tasks after fine-tuning parameters. Our main contributions are as follows:

• We develop a universal light field backbone for pixel-level structural segmentation, named LF-GPE. It utilizes a VQGAN to align epipolar plane features within the light field into a unified visual space. Meanwhile, by freezing the patch embeddings and fine-tuning the aligned light field features, our method can interactively extract both appearance and geometric structural cues from the light field.

• To enhance the generalization ability of the light field backbone in downstream tasks, we introduce LF-PMP as a generalized light field pretraining network. The proposed pretraining network prioritizes the consideration of geometric structural properties of light fields through self-supervised learning, enabling the light field backbone to accumulate a wealth of prior knowledge.

• We conduct extensive experiments on light field saliency detection and semantic segmentation datasets, confirming the effectiveness of our approach through multiple evaluation metrics. Notably, our light field modeling approach unifies the extraction of features from light fields, serving as a pioneering framework for various visual tasks based on light fields.

The subsequent sections of this paper are structured as follows. In the “Related Work” section, we present a comprehensive review of light field-related tasks, alongside an introduction to generative models and MIM. The “Methods” section outlines the design principles that underlie our proposed method. The outcomes of our experiments and a thorough analysis are presented in the “Results” section. In conclusion, the “Discussion” section summarizes the key findings of this paper and discusses potential avenues for future research.

## Related Work

### Light field in computer vision

Compared to traditional imaging, light field imaging technology provides additional 4D spatial-angular cues, allowing for its extensive use in computer vision [15,26–31], which includes salient object detection and semantic segmentation [3,4,25]. Feng et al. [24] used unsupervised methods to obtain noisy labels and integrated light field cues through joint optimization. Sheng et al. [5] introduced a challenging urban light field dataset, developing two semantic segmentation networks for effective light field cue utilization. Additionally, there is significant exploration of connections between different light field views. Zhang et al. [18] proposed a graph network-based method for efficient light field salient object detection, while Chen et al. [17] focused on sparse view input patterns and depth cues for light field saliency. Cong et al. [6] introduced a light field semantic segmentation method with an attention mechanism that leverages inter-view correlations, and Li et al. [14] designed a multi-view semantic information guided network to enhance central view features through adaptive multi-view probability fusion. However, the above methods are designed with task-specific feature extractors and lack a unified light field backbone. Additionally, these methods often sever the connection between light field features and 2D features, overlooking their correlation. In contrast, our work employs light field features as visual cues to establish their correlation with 2D features and proposes a universal light field backbone for pixel-level structural segmentation.

### Generative models

In recent years, deep generative models for image synthesis have made significant advancements. These models, starting with variational autoencoders (VAEs) [32,33] and GANs [20,34], have become essential tools for unsupervised data representation learning. One notable generative model paradigm is based on the VQGAN [21]. In contrast to conventional image generation models, VQGAN employed a codebook to discretely encode intermediate features within the model and utilized a Transformer as the encoding and generation tool. Additionally, Yu et al. [35] developed ViT-VQGAN based on the vision transformer (ViT) [11] to obtain latent codes and then applied autoregressive generation within the latent space. Chang et al. [36] introduced MaskGIT, which learned to predict tokens under random masks by attending to tokens in all directions, proposing a novel image synthesis paradigm that utilized a bidirectional Transformer decoder. While generative models have demonstrated substantial efficacy in computer vision, their exploration within the domain of light field vision remains relatively unexplored. In this paper, we try to implement a VQGAN designed to align light field features with a visual semantic space through discrete feature representations.

### Masked image modeling

Inspired by the use of BERT [37] for masked language modeling, MIM has emerged as a popular pretext task for visual representation learning [23,38]. Xie et al. [39] introduced SimMIM, a method that randomly masks a portion of the input image and predicts the original pixel values of the masked patches using an encoder-decoder. In contrast, He et al. [40] proposed MAE, which only takes visible patches as input to the encoder, significantly reducing computational overhead with this asymmetric design. For forming hierarchical visual features, Liu et al. [22] proposed MixMAE, using a mixed image as the encoder’s input. Subsequently, dual reconstruction is applied to the mixed input, resulting in a significant efficiency improvement. Li et al. [19] made a groundbreaking contribution by introducing GAN into MIM and developing MAGE, which unifies generative image modeling and representation learning within a single framework. At present, image pretraining algorithms based on MIM have demonstrated formidable potential in unsupervised learning. However, the high-dimensional structure of light fields constrains the extension of the generic MIM paradigm. In this paper, we address this issue by introducing light field structure reconstruction within the MIM framework, effectively facilitating the learning of geometric structural features in light fields.

## Results

### Implementation details

#### Datasets

In the pretraining stage, we explore various light field datasets to pretrain our LF-GPE model, including MAC [43], DUTLFMV [44], HFUTLytro [45], and Urban-Syn [5]. For fine-tuning on salient object detection, we use the DUTLF-V2 [13] dataset for training and evaluation. For semantic segmentation fine-tuning, we use the Urban-Real [5] datasets. It is worth noting that UrbanLF-Syn and UrbanLF-Real are two distinct datasets. UrbanLF-Syn is created by Blender using the Cycles and Eevee renderer, while UrbanLF-Real is a real-world dataset collected with the Lytro Illum light field camera. Moreover, our evaluation is based on the validation set from UrbanLF-Real, as the ground truth for the test set is not available.

#### Pretraining and fine-tuning

For LF-PMP pretraining, we conduct pretraining for 600 epochs. The angular resolution of the light field is set at 5 × 5, with spatial resolution set at 224 ×224. For fine-tuning on SOD, we use the SegFormer [46] framework with our pretrained LF-GPE as its backbone. All experiments are conducted using the AdamW optimizer, and the model is trained for 20 epochs with binary cross-entropy (BCE) loss. For fine-tuning on semantic segmentation, we use the UperNet [47] framework with our pretrained LF-PIT as its backbone. The training is conducted on two graphics processing units (GPUs) using the stochastic gradient descent (SGD) optimizer for a total of 160,000 training iterations, with the loss function set to cross-entropy.

### Main results of experiment

To comprehensively assess the performance of our proposed method in salient object detection, we compared it with 15 state-of-the-art 2D, 3D, and 4D salient object detection methods. These methods include five 2D RGB approaches, four 3D RGB-D methods, and six 4D light field methods. We utilize five metrics to evaluate the performance of our method, including mean absolute error (MAE), S-measure (*S*_α_) [48], E-measure (*E*_S_) [49], F-measure (*F*_β_) [50], and weighted F-measure ($F_{\beta }^{w}$) [51]. Table 1 presents detailed comparative results, clearly illustrating the superiority of our proposed model over other state-of-the-art methods. Despite the latest LFTransNet also employing Transformer for mining light field features and achieving precise salient object detection, our method still demonstrates considerable performance enhancement. In general, light field-based methods can yield promising results by leveraging implicit information within the light field images. However, they often require the design of intricate light field feature extraction modules and simultaneous training with 2D backbones. Furthermore, they struggle to effectively align the light field features with the visual semantic space. In contrast, our method introduces generative models and the LF-PMP pretraining method. This approach not only extracts multi-scale appearance features from 2D images without the need for parameter training but also integrates appearance and light field information into a unified visual semantic space using light field data.

**Table 1.** Quantitative comparisons on salient object detection with DUTLF-V2 [13]. The best results are marked in bold.

Type	Methods	Years	*MAE*↓	*S*_α_↑	*E*_S_↑	*F*_β_↑	$F_{\beta }^{w}↑$
2D	PurNet [58]	TIP21	0.059	0.848	0.883	0.815	0.771
	TRACER [59]	AAAI22	0.061	0.844	0.891	0.816	0.773
	PoolNet+ [60]	TPAMI22	0.070	0.827	0.869	0.000	0.724
	BBRF [54]	TIP23	0.051	0.869	0.905	0.000	0.820
	MENet [55]	CVPR23	0.053	0.857	0.884	0.000	0.792
3D	CCAFNet [61]	TMM21	0.047	0.856	0.903	0.825	0.784
	DFM-Net [62]	ACMMM21	0.058	0.852	0.901	0.820	0.761
	RD3D+ [53]	TNNLS22	0.057	0.843	0.881	0.800	0.740
	LSNet [63]	TIP23	0.086	0.764	0.837	0.702	0.629
4D	PANet [64]	TCyB21	0.047	0.863	0.907	0.833	0.784
	DLGLRG [65]	ICCV21	0.046	0.860	0.902	0.822	0.046
	OBGNet [66]	ACMMM21	0.038	0.900	0.940	0.890	0.840
	ESCNet [67]	TIP22	0.041	0.882	0.931	0.852	0.818
	LFBCNet [4]	ACMMM22	0.041	0.885	0.936	0.871	0.821
	LFTransNet [52]	TCSVT23	0.036	0.896	0.936	0.878	0.843
	Ours	-	**0.028**	**0.908**	**0.958**	**0.904**	**0.882**

**Fig. 2.**
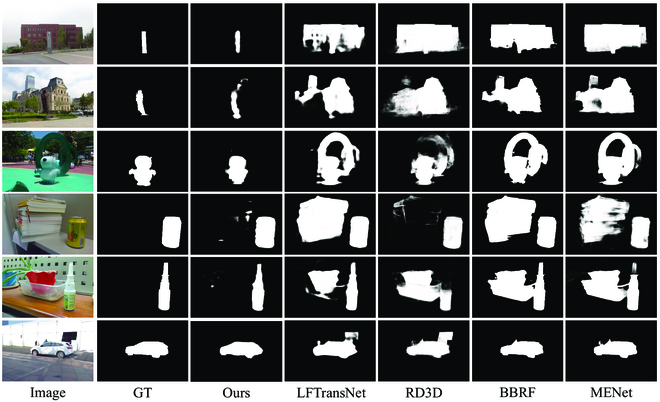
Qualitative comparisons of the state-of-the-art 2D, 3D, and 4D algorithms with our approach in some complex scenes. From the first to the last column, we show the central views of light fields, the ground truths, the saliency maps obtained form the proposed method, LFTransNet [52], RD3D [53], BBRF [54], and MENet [55].

Moreover, in Fig. 2, we provide visual comparisons of the various methods in different scenarios. These comparisons showcase complexities in different aspects, such as similar distractors, and ambiguously defined boundaries. These results intuitively demonstrate clearer outlines and more comprehensive regions in our method, emphasizing the superiority of our proposed method.

**Fig. 3.**
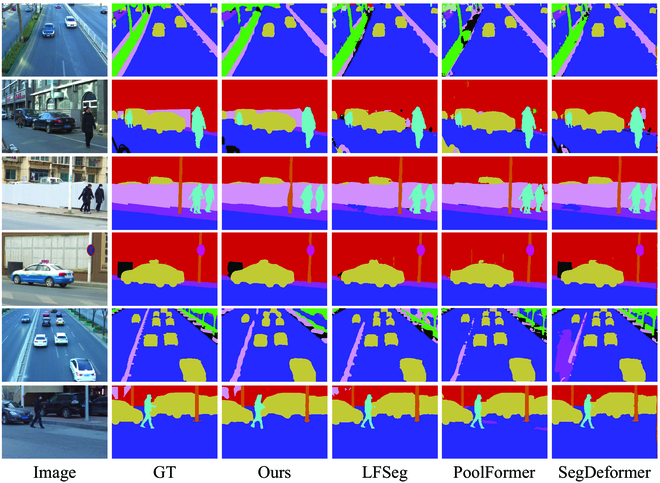
Qualitative comparisons on Urban-Real [5] validation set for semantic segmentation. From the first to the last column, we show the central views of light fields, the ground truths, the segmentation maps obtained form the proposed method, LFSeg [25], PoolFormer [56], and SegDeformer [57].

Furthermore, we compared our proposed method with other methods in the task of semantic segmentation. Due to the limited research and availability of open-source code for light field semantic segmentation, we only compared our method with six other state-of-the-art semantic segmentation methods. These include four image-based methods and two light field-based methods. These methods represent a comprehensive selection and are accompanied by open-source code. Our evaluation employs three key metrics: pixel accuracy (Acc), mean pixel accuracy (mAcc), and mean intersection over union (mIoU). The quantitative comparison results between our method and others can be found in Table 2. By integrating our proposed LF-GPE as a modification to the backbone of UperNet, we have significantly improved the effectiveness of semantic segmentation. Our method achieves the highest scores across almost all metrics. The qualitative results of our approach are illustrated in Fig. 3.

**Table 2.** Quantitative comparisons on semantic segmentation with Urban-Real [5]. The best results are marked in bold.

Type	Methods	Years	Acc	mAcc	mIoU
Image-based	SegDeformer [57]	ECCV22	90.85	82.25	73.50
	Swin-Transformer [12]	ICCV21	92.22	83.38	76.42
	ResNeSt [68]	CVPR22	90.98	82.94	76.16
	PoolFormer [56]	CVPR22	91.39	80.91	74.84
Light field-based	LF-IENet [6]	CVPR23	93.19	85.55	76.28
	LFSeg [25]	TIM21	90.72	83.80	74.48
	Ours	-	**93.45**	**86.33**	**76.79**

### Ablation study

In this section, we conducted an ablation analysis on the light field salient object detection dataset DUTLF-V2 [13] to validate the effectiveness of each component of our proposed method.

#### Effectiveness of architecture design

To validate the effectiveness of the proposed architecture, we modify our model into different variants. As shown in Table 3, among them, “2D” represents the usage of only the central view of the light field as input. “LFPE” represents the exclusion of the VQGAN for quantizing input epipolar plane views into semantic tokens, thereby not aligning the light field features into a unified visual semantic space. “LF-GPE ^∗^” represents the omission of computing the difference in these semantic tokens, merely concatenating the semantic tokens together. Compared to the 2D method, the incorporation of light field visual cues has significantly enhanced the evaluation metrics of these methods. This demonstrates that light field visual cues can provide richer geometric structural information for visual models, thereby facilitating pixel-level segmentation results. Compared to LFPE, our proposed method significantly improves performance, demonstrating the effectiveness of the generative model. LF-GPE, as proposed by us, effectively utilizes the implicit discrete feature space within the generative model to align light field features into a unified visual semantic space. Simultaneously, we observe that removing the computation of semantic token differences and solely concatenating the semantic feature leads to a degradation in the performance of the saliency detection model. The primary reason for this issue lies in the redundant light field information across different views, where simple feature concatenation makes it challenging for the network to effectively leverage valuable light field features with a limited number of parameters. In addition, we also compared the performance of our method under different initialization parameters. The results demonstrate that our proposed light field pretraining method significantly enhances the model’s accuracy in downstream tasks.

**Table 3.** Quantitative comparisons on salient object detection with different variants. The best results are marked in bold.

Methods	Initialize weights	Param (M)	Fully train	Fine-tune	*MAE*↓	*S*_α_↑	*E*_S_↑	*F*_β_↑	$F_{\beta }^{w}↑$
2D	MixMIM	91.4			0.044	0.869	0.927	0.852	0.813
LFPE	MixMIM	105			0.043	0.871	0.928	0.857	0.817
LFPE	MixMIM	19.0			0.042	0.880	0.932	0.864	0.848
LF-GPE	^∗^MixMIM	99.3			0.043	0.869	0.945	0.878	0.841
LF-GPE	^∗^MixMIM	12.3			0.035	0.889	0.840	0.875	0.875
LF-GPE	MixMIM	97.8			0.044	0.867	0.927	0.852	0.811
LF-GPE	MixMIM	10.8			0.032	0.899	0.950	0.890	0.853
LF-GPE	LF-PMP	97.8			0.041	0.876	0.932	0.862	0.825
LF-GPE (our)	LF-PMP	10.8			**0.028**	**0.908**	**0.958**	**0.904**	**0.882**

**Fig. 4.**
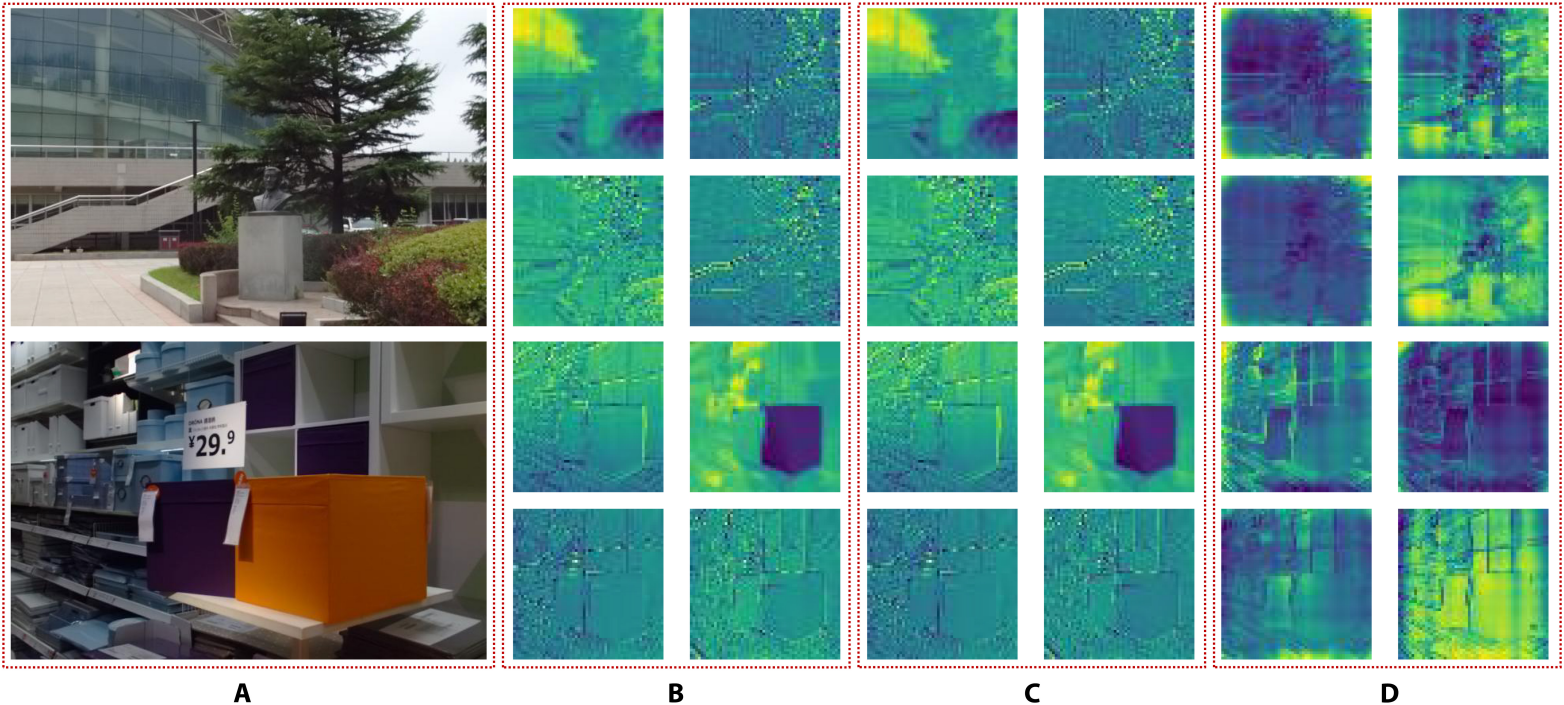
The intermediate feature maps of the first-layer neurons in our method. (A) Central view of the light field. (B) Feature maps before incorporating light field prompting. (C) Feature maps after incorporating light field prompting. (D) Light field prompting feature maps.

**Fig. 5.**
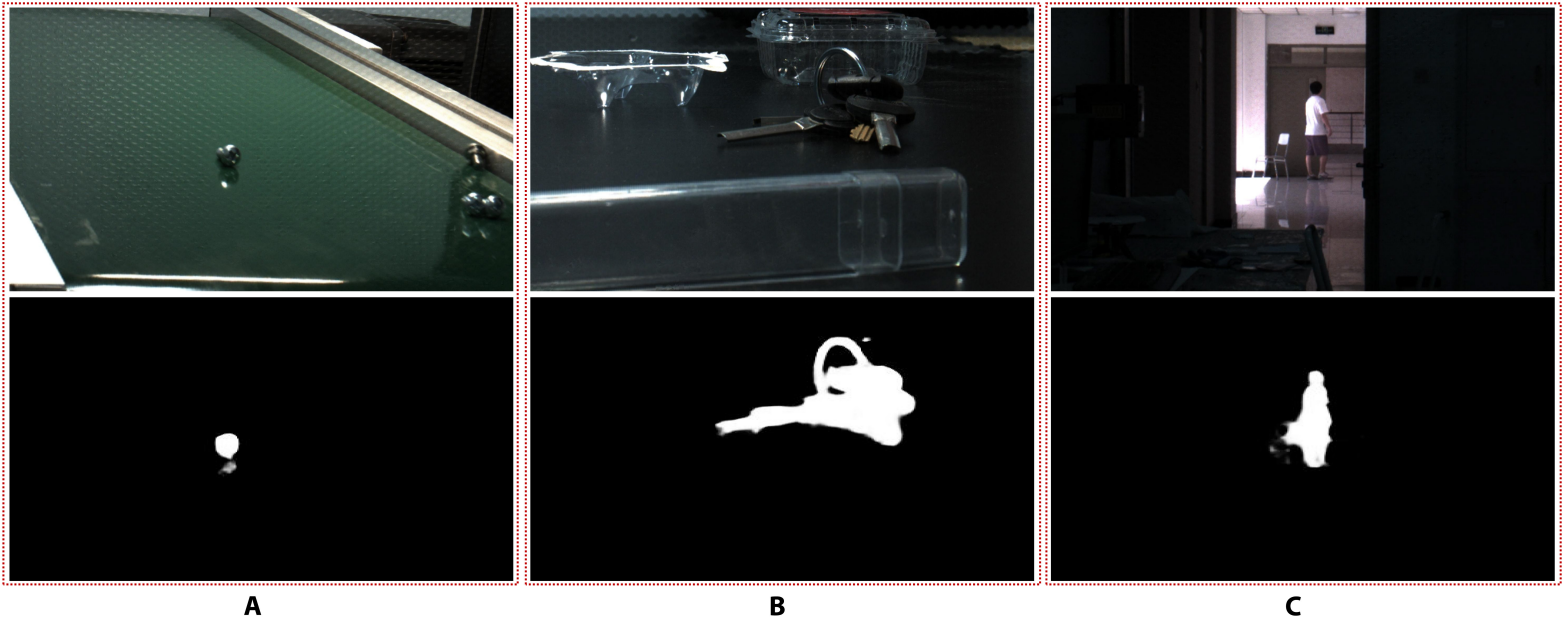
(A to C) The visualization examples of our method in real scenarios. Top: Central views of light fields captured in real situations with the Raytrix R8 light field camera. Bottom: Final saliency maps produced by our method.

In addition to quantitative metrics, we are also conducting some qualitative analyses. As shown in Fig. 4, we visualize the intermediate feature maps of the first-layer neurons in our method. Here, (A) represents the central view of the light field, (B) and (C) represent the feature maps before and after incorporating light field prompting, and (D) represents the light field prompting feature maps. Visually, there is no apparent difference between (B) and (C). However, fundamentally, the feature map (C) is the result of integrating the feature map (B) with the light field prompting (D). From the light field prompting feature map, we can observe that the geometric structural information brought by the light field primarily distributes at the edges of objects, confirming that the most prominent light variations in the light field originate from the regions where there are abrupt changes in the object structure. Therefore, in our method, we effectively describe the geometric structural features of the light field by removing redundant appearance features and retaining light field clues from the object boundaries through the computation of different light field semantic token differences. Furthermore, we extend the proposed method to real scenarios. Specifically, we use the Raytrix R8 light field camera to capture light field videos in three different scenarios, extracting relevant frames for light field SOD predictions. The results, depicted in Fig. 5, showcase the excellent performance of our proposed method in practical environments.

#### Performance and complexity analysis

We attempt to answer the question: Does visual prompting truly improve the efficiency of light field training and the accuracy of inference? Thus, we showcase the performance of our proposed method by recording the trainable parameters of different methods under both fully train and fine-tune. The results are shown in Table 3. These results indicate that fine-tune outperforms fully train, attributing this to the ability to inherit prior RGB knowledge by freezing the parameters of the RGB model, thus demonstrating the superiority of light field prompting. Furthermore, the number of parameters required to fine-tune is significantly less than that to fully train. Our proposed LF-GPE only needs to train 10 million parameters to achieve optimal performance.

#### Hyperparameter analysis

We introduce *λ* in light field feature interactive modeling to control the dimension of learnable features. A larger *λ* reduces the feature dimension of the interaction between the light field and appearance features, allowing for fine-tuning with fewer parameters. As shown in Table 4, the model’s performance improves as *λ* decreases from 16 to 2. However, when *λ* continues to decrease to 1, even with an increased model size, it fails to consistently achieve better performance. This suggests that *λ* = 2 is a reasonable choice for balancing performance and model size.

**Table 4.** Quantitative comparisons on hyperparameters with *λ* in the context of light field feature interactive modeling. The best results are marked in bold.

	Param (M)	*MAE*↓	*S*_α_↑	*E*_S_↑	*F*_β_↑	$F_{\beta }^{w}↑$
*λ* = 1	18.9	0.032	0.898	0.951	0.889	0.850
*λ* = 2	10.8	**0.028**	**0.908**	**0.958**	**0.904**	**0.882**
*λ* = 4	8.1	0.301	0.904	0.955	0.897	0.865
*λ* = 8	7.1	0.305	0.902	0.953	0.896	0.861
*λ* = 16	6.6	0.317	0.899	0.949	0.890	0.855

## Discussion

In this paper, we introduce a comprehensive framework for modeling light field features, comprising an LF-GPE and an LF-PMP network. Specifically, LF-GPE can serve as a backbone for light fields, requiring minimal parameter training to simultaneously extract both appearance and geometric structural information from the light field. Crucially, our proposed LF-GPE aligns the light field features within a universal visual semantic space, thereby generating generalized visual cues tailored for light fields. Furthermore, the LF-PMP serves as a generalized pretraining network for light fields, utilizing mask mechanisms to drive LF-GPE in extracting comprehensive geometric structural information. Finally, we evaluate the effectiveness of our approach in two downstream tasks: light field salient object detection and semantic segmentation. The results demonstrate the capability of our proposed method to accurately segment objects from complex backgrounds. Moreover, numerical analyses confirm the highly competitive performance of our proposed light field modeling approach in these pixel-level segmentation tasks.

The work presented in this paper marks a pivotal milestone in the application of GAI based on light fields, contributing to the development of both virtual and real-world scenarios and catalyzing progress in autonomous driving. Furthermore, the development of light field feature modeling methods establishes a new foundation for expansion into machine vision. However, it is crucial to note that the LF-GPE model proposed in this paper relies on transformer-based modeling for light field features, incurring substantial computational costs. Therefore, in the future, we plan to conduct additional research on lightweighting light fields for deployment on edge devices.

## Methods

### Preliminary

We first revisit the recent 2D RGB-based backbone, Swin Transformer [12], which achieves significant pixel-level predictions by encoding multi-scale visual representations. Moreover, it can encode data-related features in MIM [22,39]. The input to the backbone is a 2D image *I*(*x*, *y*) ∈ ℝ^*H*×*W*×3^. It is first divided into *N* nonoverlapping patches using a patch cropping module, with each patch having a resolution size of *P* × *P*. Here, *N* is calculated as *N* = *H* × *W*/*P* × *P*. Then, a linear projection is applied to these image patches to generate patch embeddings *E*. Next, a learnable positional embedding is incorporated into *E* to embed spatial information. Afterward, all of these patches are concatenated into a sequence of length *N* and fed into an encoder. Each encoder layer updates the input patches using multi-head self-attention modules with either regular windowing (W-MSA) or shifted windowing (SW-MSA), along with a feed-forward network (FFN). To avoid symbol misuse, we will refer to both W-MSA and SW-MSA simply as MSA, as they both denote multi-head self-attention based on window partitioning. Moreover, to generate a feature pyramid, each encoder layer conducts feature map downsampling by merging patches. For instance, in the first encoder layer, the features of every group of 2 × 2 adjacent patches are merged, and a linear layer is applied to the concatenated features. This reduction leads to a fourfold decrease in the number of patches, equivalent to a twofold downsampling in resolution. Formally, the feature modeling of the *l*-th encoder layer can be described as follows:

$$q=k=v=E^{l}$$ (1)

$$E^{\prime }=E^{l}+\mathrm{MSA}\left(q,\;k,\;v\right)$$ (2)

$$E^{l+1}=M\left(E^{l}+\mathrm{FFN}\left(E^{\prime }\right)\right)$$ (3)

where *E* represents the input feature sequence for the *l*-th encoder layer. We use *q*, *k*, and *v* to denote the queries, keys, and values input into the multi-head window attentionm respectively. *M* represents the patch merging operation. Since the flexibility of this pyramid structure allows for modeling at different scales and has linear computational complexity with respect to image size, the 2D RGB-based backbone efficiently conducts image feature modeling. Finally, the output of each layer is reshaped into feature maps and used as input for prediction heads in downstream tasks.

### Generative light field prompting encoder

**Fig. 1.**
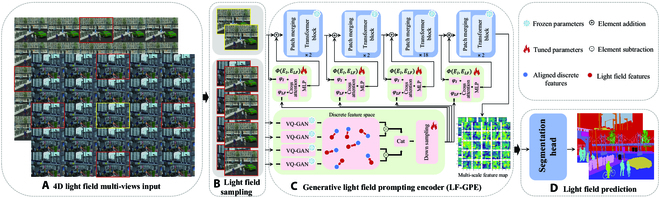
An overview of LF-GPE. (A) Multi-view matrices of the light field. (B) Light field sampleing from multi-view matrices. (C) LF-GPE. (D) Light field segmentation prediction head.

Recently, prompt learning has emerged, aiming to enhance the performance of visual tasks by introducing prompt information to the input without significantly altering the structure and parameters of pretrained models [41,42]. Inspired by this, as shown in Fig. 1, we extend the Swin Transformer [12] and VQGAN [21] to 4D light fields prompting and introduce LF-GPE.

Specifically, given a 4D light field *L*(*u*, *v*, *x*, *y*) ∈ ℝ^*U*×*V*×*H*×*W*×3^ obtained through a parameterized two-model, we first acquire the central view $L_{I}\left(u=\frac{U}{2},v=\frac{V}{2},x,y\right)$ and the epipolar plane views $L_{\mathrm{up}}\;\left(u\;=\;\frac{U}{2},v\;=\;\frac{V}{2},x,y\right),L_{\text{down}}\;\left(u\;=\;U,v\;=\;\frac{V}{2},x,y\right),$ $\left.L_{\text{left}}\left(u=\frac{U}{2},v=0,x,y\right),L_{\text{right}}\left(u=\frac{U}{2},v=V,x,y\right)\right\}$. It is worth noting that these epipolar plane views provide a description of light field geometric properties from the epipolar plane, making it suitable for extracting light field features. Second, we divide the central view into *N* nonoverlapping patches and conduct linear projection to generate patch embeddings *E*_I_ Third, we employ a VQGAN [21] to quantize input epipolar plane views into semantic tokens. To enhance the representation information within the light field, we further compute the difference in these semantic tokens, as formulated below:

$$E_{\mathrm{LF}}=\mathrm{cat}\left(\left|\mathrm{GAN}\left(L_{\mathrm{up}}\right)-\mathrm{GAN}\left(L_{\text{down}}\right)\right|,\right.\left.\left|\mathrm{GAN}\left(L_{\text{left}}\right)-\mathrm{GAN}\left(L_{\text{right}}\right)\right|\right)$$ (4)

where *GAN* represents the encoder of a VQGAN and *cat* denotes the concatenation operation. Due to the codebook in the VQGAN, which can transform inputs into unified visual spaces, the semantic tokens generated from the light field can be effectively aligned with appearance visual features from the central view. Then, *E*_LF_ is downsampled through patch merging to create a multi-scale light field prompt ${\left\{{\hat{E}}_{\mathrm{LF}}\right\}}_{\;l=1}^{\;N}$, where *N* represents the number of scale layers. Following this, we introduce a trainable light field interaction module before each encoding layer and employ an additive operation to merge features. The interactive modeling rules for the light field of the *l*-th encoder layer are defined as:

$$E^{l}=E^{l}+\Phi \left(E_{I}^{l},{\hat{E}}_{\mathrm{LF}}^{\;l}\right)$$ (5)

$$\Phi \left(E_{I},E_{\mathrm{LF}}\right)=\mathrm{MLP}\left(\text{Cross}\left(\varphi_{I}\left(E_{I}\right),\varphi_{\mathrm{LF}}\left(E_{LF}\right)\right)\right)$$ (6)

where Φ represents the interactive modeling, consisting of two linear projections, denoted as *φ*_I_ and *φ*_LF_, a cross-attention operation, and an *MLP* unit. It is worth noting that in *φ*_I_ and *φ*_LF_, we control the scaling of feature dimensions by setting a scale factor *λ*. Additionally, during the training process, only the parameters of the light field interaction module Φ are fine-tuned. Finally, the generated feature *E^l^* is fed into the next encoder layer to produce multi-scale features. In this way, our proposed LF-GPE not only extracts multi-scale appearance features from 2D images without the need for parameter training but also aligns the appearance and light field information into a unified visual semantic space using light field data.

### Prompt-based masked light field pretraining

While LF-GPE is effective in aligning light field features, the randomly initialized parameters of the interaction model currently hinder its ability to model the geometric structure of the light field. Furthermore, utilizing traditional MIM for pretraining LF-GPE is inefficient due to the limited availability of light field datasets. Therefore, in this work, we leverage the core concept of visual prompt fine-tuning and introduce a comprehensive light field pretraining network known as LF-PMP. The detailed description of LF-PMP Training algorithm pipeline in pseudocode follows.


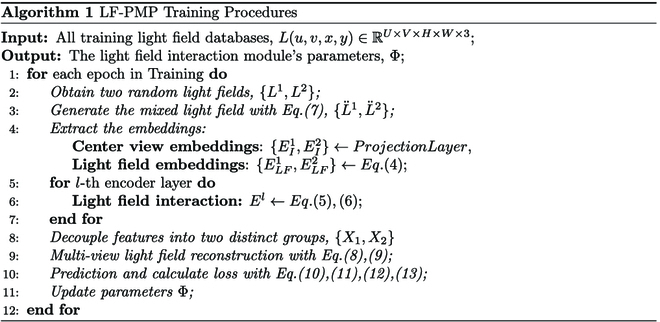


### Light field prompt for encoding

This module aims to fine-tune light field interaction module Φ in LF-GPE. Following the same method as in [22], we first create the mixed light field $\left\{{\ddot{L}}^{1}\;,\;{\ddot{L}}^{2}\right\}$ from two sets of random training light fields {*L*^1^, *L*^2^}. This process is expressed as:

$${\ddot{L}}_{I}^{1}=L_{I}^{1}⊙M+L_{I}^{2}⊙\left(1-M\right)$$ (7)

where *M* is defined as the mask symbol and ${\ddot{L}}_{I}^{2}$ is generated using the same operations. Then, we obtain the central view embeddings $\left\{{\hat{E}}_{I}^{1},{\hat{E}}_{I}^{2}\right\}$ and the light field embeddings $\left\{{\hat{E}}_{\mathrm{LF}}^{1},{\hat{E}}_{\mathrm{LF}}^{2}\right\}$ through the patch embedding layer of LF-GPE. Finally, we input the generated embeddings into the light field interaction module and other layers for pretraining. Specifically, LF-PMP is initialized with pretrained parameters from Swin Transformer, and the light field interaction module Φ in each layer is designated as a trainable neural layer, while the other layers are frozen. The effective mixing and masking operations intentionally obscure a substantial portion of visible information within the light field. This compels the light field interaction module Φ to enhance interactions among visible features, enabling it to infer the distribution of hidden features and thereby improving network learning efficiency.

### Multi-view light field reconstruction

For light field reconstruction, we employ a multi-view light field structure reconstruction method distinct from 2D MIM [23]. First, after obtaining the encoded multi-scale light field features, we decouple these features into two distinct groups using the binary mask *M*. Following this, we utilize a decoder comprising eight transformer blocks with an embedding dimension of 512 to reconstruct the original light fields for both groups. These reconstructed features are defined as *F* = {*X*_1_, *X*_2_}. To reconstruct the complete topological structure of the light field, we leverage light field prompt *E*_LF_ to decode four distinct structural deviations. Finally, we use the reconstructed features *F* and the structural deviations ${\hat{E}}_{\mathrm{LF}}$ to reconstruct both the central view and the light field epipolar plane views. For the former, we apply the traditional MIM method for pixel-wise reconstruction, while for the latter, we incorporate the learned structural deviations into the original output features *F* in a residual manner to achieve structural reconstruction. This process can be defined as follows:

$$\hat{I}=\Psi_{A}\left(F\right)$$ (8)

$${\hat{L}}_{*}=\Psi_{\mathrm{LF}}\left(F+D_{*}\left({\hat{E}}_{\mathrm{LF}}\right)\right)$$ (9)

where Ψ_A_ and Ψ_LF_ respectively represent the light field appearance and structure reconstruction heads. *D*_∗_ represents the light field structural deviation encoding, which is composed of multiple multi-layer perceptrons (MLPs) with different light field directions. The total loss can be formulated as:

$$L_{\mathrm{rec}}=L_{I}+L_{H}+L_{V}$$ (10)

$$L_{I}={\left\|\left(L_{I}^{1}-{\hat{I}}^{1}\right)⊙\left(1-M\right)\right\|}_{2}^{2}+{\left\|\left(L_{I}^{2}-{\hat{I}}^{2}\right)⊙M\right\|}_{2}^{2}$$ (11)

$$L_{H}={\left\|\left(L_{H}^{1}-{\hat{L}}_{H}^{1}\right)⊙\left(1-M\right)\right\|}_{2}^{2}+{\left\|\left(L_{H}^{2}-{\hat{L}}_{H}^{2}\right)⊙M\right\|}_{2}^{2}$$ (12)

$$L_{V}={\left\|\left(L_{V}^{1}-{\hat{L}}_{V}^{1}\right)⊙\left(1-M\right)\right\|}_{2}^{2}+{\left\|\left(L_{V}^{2}-{\hat{L}}_{V}^{2}\right)⊙M\right\|}_{2}^{2}$$ (13)

where ${\hat{I}}^{1}$ and ${\hat{I}}^{2}$ represent the reconstructed central views corresponding to $L_{I}^{1}$ and $L_{I}^{2}$, respectively. ${\hat{L}}_{H}^{1}=\left({\hat{L}}_{\text{left}}^{1}-{\hat{L}}_{\text{right}}^{1}\right)$ and ${\hat{L}}_{V}^{1}=\left({\hat{L}}_{\mathrm{up}}^{1}-{\hat{L}}_{\text{down}}^{1}\right)$ represent the structural differences of the light field in the horizontal and vertical directions, respectively. Additionally, the ground truth for the structural differences is computed from the epipolar plane views. The underlying insight is that, given that the epipolar plane views encapsulate the most prominent structural changes within the light field, we can model the geometric structure of the light field by reconstructing the difference between these views.

## Data Availability

The datasets generated and analyzed during the current study are available from the corresponding author upon request.
